# The Impacts of Burn Severity and Frequency on Erosion in Western Arnhem Land, Australia

**DOI:** 10.3390/s24072282

**Published:** 2024-04-03

**Authors:** David Bretreger, Gregory R. Hancock, John Lowry, Indishe P. Senanayake, In-Young Yeo

**Affiliations:** 1 College of Engineering, Science and Environment, The University of Newcastle, Callaghan, NSW 2308, Australia; david.bretreger@uon.edu.au (D.B.); indishe.senanayake@newcastle.edu.au (I.P.S.); in-young.yeo@newcastle.edu.au (I.-Y.Y.); 2 Ecosystem Restoration and Landform Team, Environmental Research Institute of the Supervising Scientist (ERISS), Darwin, NT 0820, Australia; john.lowry@dcceew.gov.au

**Keywords:** Northern Territory, fire, surface erosion, soil, burn severity, remote sensing, *dNBR*

## Abstract

Wildfires are pivotal to the functioning of many ecosystems globally, including the magnitude of surface erosion rates. This study aims to investigate the relationships between surface erosion rates and wildfire intensity in the tropical north savanna of Australia. The occurrence of fires in western Arnhem Land, Northern Territory, Australia was determined with remotely sensed digital datasets as well as analogue erosion measurement methods. Analysis was performed using satellite imagery to quantify burn severity via a monthly delta normalised burn ratio (*dNBR*). This was compared and correlated against on-ground erosion measurements (erosion pins) for 13 years. The *dNBR* for each year (up to +0.4) displayed no relationship with subsequent erosion (up to ±4 mm of erosion/deposition per year). Poor correlation was attributed to low fire severity, patchy burning, significant time between fires and erosion-inducing rainfall. Other influences included surface roughness from disturbances from feral pigs and cyclone impacts. The findings here oppose many other studies that have found that fires increase surface erosion. This accentuates the unique ecosystem characteristics and fire regime properties found in the tropical Northern Territory. Scenarios of late dry season fires with high severity were not observed in this study and require more investigations. Ecosystems such as the one examined here require specialised management practices acknowledging the specific ecosystem functions and processes. The methods employed here combine both analogue and digital sensors to improve understandings of a unique environmental system.

## 1. Introduction

The impact of wildfires and prescribed burns on environmental assets and processes is a topic of interest globally [1]. Fires have always occurred in certain environments [2] and have the potential to cause widespread impacts [3,4,5,6,7]. Environmental factors play a big role in how fires behave, ranging from vegetation communities, topography, fuel load and to climate. While there are obvious impacts on vegetation biomass and associated ecological impacts, the subsequent environmental and ecosystem effects on erosion and streamflow are also important. It is crucial that the impact of fire can be quantified so planners and resource managers can distribute adequate resources, plan for future wildfire events and the subsequent influences on the environment. 

Fire or burn severity is a key factor associated with the ecosystem response to a fire. A higher burn severity will result in more vegetation loss, both spatially and vertically through canopy burning, and typically longer recovery times. The loss of organic matter has been shown to lead to ecosystem responses such as erosion [8]. Many studies have tried to understand if and why this is occurring, and how much erosion should be expected in a range of different ecosystems globally [9,10,11,12]. Typically, this soil loss is expected to be through land flow erosion processes. Physical vegetation loss is a contributing factor to the erosion processes, although soil chemistry is also a part of the erosion process influenced by fire. Shakesby et al. [13] summarised the changes in soil chemistry from fires, concluding that it changes the wettable area and how water repellent the top layer of soil becomes. This is thought to be a major factor impacting the erosion process after fire, which can change considerably in differing environments. 

The spatial nature of fires and the impacts on surface erosion can be difficult to quantify. While firefighting services in some regions can routinely map the impacted area of a wildfire or prescribed burn, spatial data that incorporates the severity of the burn are more difficult and time-consuming to acquire [14]. Fire-impacted area is even more difficult to obtain using the on-the-ground methods in areas with large spatial extents, low population density and problematic access. Satellite remote sensing algorithms have been developed that can assess the burn severity using optical and infrared data [15,16]. Various forms of satellite data are routinely collected globally and hence can provide before and after observations of fire impacts, albeit with observational challenges caused by smoke. As more satellites become available, the ability of these observational insights will only increase. The monitoring of vegetation recovery post-fire using remote sensing vegetation indices (i.e., NDVI or similar) is well studied [17,18]. 

The use of remote sensing in determining fire severity is particularly important in sparsely populated areas as there are not enough human resources to collect data. In such areas, remote sensing can provide information on fire location and timing [19]. In light of this, several studies have been conducted investigating bushfires and their impact on erosion by using remotely sensed data. For example, Sánchez et al. [20] studied pre- and post-fire erosion using Sentinel-2 and LiDAR data. They utilised the normalised burn ratio (*NBR*) to identify bushfire-affected areas in the Garganta de los Infiernos Nature Reserve, Spain. The erosion was quantified through multi-criteria analysis using the Revised Universal Soil Loss Equation (RUSLE). A similar approach, integrating *NBR* and RUSLE, was employed by Efthimiou et al. [21] to map the fire severity and soil erosion susceptibility by the Mati fatal wildfire in Eastern Attica, Greece. Argentiero et al. [22] used NDVI and relative differenced *NBR* to analyse the impact on fire severity on soil erosion. Mallinis et al. [23] utilised a combination of ASTER imagery, air photos and Landsat TM imagery to map pre- and post-burn severity in their study modelling post-fire erosion risk from a large and intensive wildland fire at the watershed level in a Mediterranean landscape to prioritise protection and management. Pérez-Cabello et al. [24] provided a comprehensive review on the remote sensing techniques employed in evaluating post-fire vegetation recovery, focusing on tools, recent developments, sensors and indicators/metrics. Reiner et al. [25] developed region-specific models for the American Southwest to assess the post-fire burn severity by collecting field data of 21 wildfires and developing pre- and post-fire delta *NBR*, relative delta *NBR* and the relative burn ratio using Landsat and Sentinel-2 imagery. Howe et al. [26] conducted a comparison between Landsat 8 and Sentinel-2 for burn severity mapping in western North America. They concluded that Sentinel-2 may enhance the ecological discrimination of fine-scale fire effects, attributed to its higher spatial resolution. Kurbanov et al. [27] conducted a review of various remote sensing techniques used to assess forest burnt areas, burn severity and post-fire recovery.

The Northern Territory of Australia is an example of a region that is frequently fire-affected, although due to low population numbers, fire management is difficult [28]. This region has been subject to many studies of ecological impacts of fire [29,30,31] and landscape evolution erosion [32,33,34], although a detailed study on the impacts that these fires have on surface erosion is unexplored. 

This paper examines: (1) the role of frequent burning of vegetation and its effect on surface erosion rates in a catchment representative of a post-mining landscape using analogue and digital sensors, (2) how to quantify burn severity using remote sensing images from the Landsat data to assess and quantify subsequent erosion and (3) how to demonstrate how a combination of both analogue and digital sensors can be used to better understand remote environments.

## 2. Materials and Methods

### 2.1. Site Description

**Figure 1:**
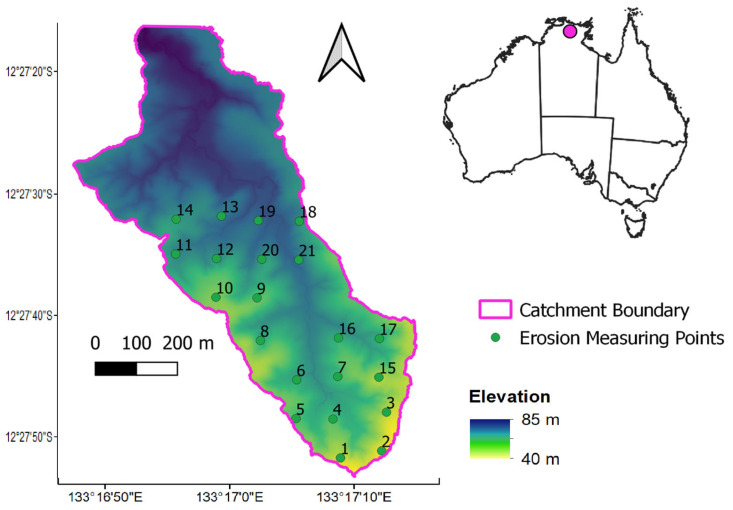
The location of study catchment with erosion measuring points shown. The coloured gradient shows the elevation from the 1 m LiDAR digital elevation model.

The study area, Tin Camp Creek catchment (~40.8 ha), is located in the Alligator Rivers Region in western Arnhem Land of Northern Territory, Australia (Figure 1). In long term, this site is believed to be comparable to the Ranger uranium mine’s rehabilitated landforms [35]. The rainfall is mostly received during the wet season, which typically spans from November to April. The Tin Camp Creek catchment received an average rainfall of 1540 mm during the wet season in the period of 1971–2020 (Source: BOM Station ID 14198—Jabiru Airport). The minimum air temperature is recorded at 22.6 °C, while the maximum reaches 34.3 °C [33].

The native vegetation consists primarily of open dry-sclerophyll forests characterised by *Eucalyptus* and *Acacia* species [36]. *Melaleuca* spp. and *Pandanus spiralus* are also present in the low-lying riparian areas, while the undergrowth is primarily comprised of *Heteropogon contortus* and *Sorghum* spp. A vigorous growth of annual grasses can be seen during the early wet season. These grasses often fall over during the wet season creating a thick mulch layer which significantly reduces the bare soil erosion. Fires frequently reduce the vegetation cover in the dry season, thereby increasing the potential of erosion through overland flow [37].

Heimsath et al. [38] identified a ‘humped’ soil-production function, with peak soil production rates occurring under a soil thickness of approximately 35 cm in the area. The landscape is erosionally stable, as per the similarity found by Hancock and Lowry [33] between the hillslope erosion and soil production. Despite these findings and minimal human interference in the landscape, there are signs of erosion susceptibility due to soil pedestals, sheet wash and soil disturbance caused by feral pigs (*Sus scrofa*) [32]. 

### 2.2. Fire Regime

Werner and Peacock [31] stated that humans are the ignition source for almost all dry season fires in the region. Indigenous people have lit these fires for various management purposes for tens of thousands of years. One of these purposes was to reduce the impact of wildfires through hazard-reduction burns. Fires started without human intervention are rare, accounting for only 4% [39]. These are attributed to dry lightning storms at the start of the wet season. Once ignited, it has been reported that fire can be spread by windblown embers and by birds, commonly referred to as ‘firehawks’, in tropical Australian savannas [40]. The fire regime is impacted by climate oscillations, including El Niño–Southern Oscillation (ENSO), which, it has been suggested, can help for seasonal predictions of fire severity [41]. There has also been an increase in the distribution of *Andropogon gayanus* (gamba grass), an invasive species, which has been shown to change fire behaviour and increase severity [42,43,44]. 

**Figure 2:**
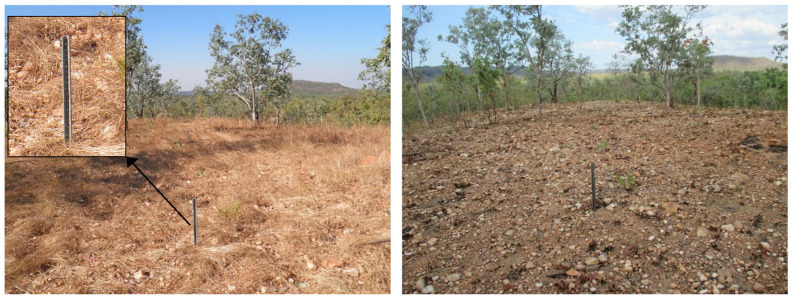
One of the erosion measurement locations showing the ‘V’-shaped dropper. The dropper extends 500 mm above the soil surface. The left photo shows conditions before burning while the right photo shows conditions following fire has been through the area. The surface displays a high rock content or armour.

Fires in the region are generally only low-level ground fires that do not scorch the tree canopies [31] (Figure 2). However, if understorey grasses are allowed to accumulate through the dry season, thereby creating a large and dry fuel load, this may result in more severe fires late in the dry season [30]. 

### 2.3. Data

#### 2.3.1. Remote Sensing Data

Remote sensing data for assessing the burn severity for this study were obtained from the Landsat constellation (i.e., Landsat 5, 7 and 8). The Landsat 5, 7 and 8 images were obtained from Digital Earth Australia (DEA) [45]. These datasets are provided as analysis-ready surface reflectance data and covers the time period considered in this study. These datasets have been processed to account for atmospheric corrections, terrain shadow, geometric distortions and pixels obscured by clouds. The spatial resolution is 30 m with a revisit time of 16 days for each satellite. Combining data from multiple Landsat missions increases the data frequency compared to the revisit time of an individual satellite. The period being assessed was from January 2005 to December 2018 to coincide with the erosion measurements, resulting in 531 observations being available. The individual satellite observations are aggregated to a monthly mean to create a gap-filled time series, and hence there is 1 value per month made up of 2–4 images depending on which Landsat satellites were operational at the time. The monthly mean composites mitigate the stripes caused by the SLC sensor malfunction on Landsat 7 after May 2003. The script used in DEA to specify the data to be used is provided in the Supplementary Materials for this article.

The fire occurrence dates were obtained from North Australia and Rangelands Fire Information (NAFI, https://firenorth.org.au/, accessed on 21 March 2024), which provides monthly details of spatial fire occurrence at ~250 m resolution based on a range of satellite information sources. 

Additional remote sensing data were used in the form of a 1 m Light Detection and Ranging (LiDAR)-derived DEM of the catchment. These were downloaded as a LiDAR point cloud and processed to a DEM for the catchment using LasTools (version 190623). The elevation data are available from Geoscience Australia from the ELVIS spatial data catalogue (https://elevation.fsdf.org.au/, accessed on 21 March 2024). These data were used to assess various terrain features (i.e., topographic wetness index (TWI) [46] and SAGA Wetness Index (SWI) [47]) with the erosion measurements. The TWI and SWI give an indication of catchment relative wetness based on topography and locations where runoff accumulates [48].

#### 2.3.2. In Situ Erosion Measurements

Erosion measurements were derived from erosion pins and droppers that involve measuring the change in surface soil height on a metal pin or dropper placed on the hill slope. As erosion represents a decrease in soil level, its measurement is reflected as a negative value. Conversely, deposition is noted when the pin shows a reduction in measured exposure. The pin and dropper measurements are described in detail by Hancock and Lowry [33] and are a well-recognised and simple way of reliably measuring surface erosion rates and deposition [49]. The locations of these points are presented in Figure 1 with annual data available from 2005 to 2018. The erosion pins are 300 mm long and 5 mm in diameter, with 50 mm initially exposed above the surface. The small diameter of the erosion pins means they cause minimal disruption of natural rainfall-runoff processes while mimicking the predominant grass in the region. The droppers are metal stakes 810 mm long and 50 mm wide. They are installed with 500 mm exposed for erosion measurements. They are a V-shaped in cross-section, which is inserted with the ‘V’ facing upslope. The shape of the products mean extra deposition can be collected. A photo showing the installation of one of the V-shaped droppers is included as Figure 2. The initial placement of these measurement sites was recorded with a differential GPS for accurate positioning. The erosion measurements are made annually using a Vernier calliper to record the length of the exposed pin or a rigid rule for the dropper. If erosion measurement was missed in one year, the change in soil height measured the following year was averaged to provide each of the two required annual figures. Erosion measurements were recorded in the dry season between June and October when erosive rainfall is typically minimal or absent. Erosion measurements were recorded in all years except 2010 and 2014.

### 2.4. Methodology

The analysis-ready (pre-processed) remote sensing data have some missing observations. To provide a more comprehensive spatial coverage over the catchment, a monthly mean of all available observations was calculated. To account for slight surface reflectance differences, the Landsat 8 surface reflectance was corrected to match the surface reflectance of Landsat 7 via the equations developed specifically for Australia by Flood [50]. There were still some minor sections of the catchment not covered with observations, although these had negligible effect on the analysis. 

To assess burn severity via remote sensing the *NBR* [16,51] was used. The *NBR* is defined as:

(1) $$NBR=\frac{\left(NIR-SWIR2\right)}{\left(NIR+SWIR2\right)}$$

where NIR is the near infrared band (i.e., 770–900 nm for Landsat 7 ETM+) and SWIR2 is the shortwave infrared band 2 (i.e., 2090–2350 nm for Landsat 7 ETM+). The *NBR* provides a unitless value between −1 and 1. Healthy green vegetation will have a higher *NBR* value, while burnt vegetation will have a low value. Areas of dry, brown vegetation or bare soil will also return lower *NBR* values than green vegetation. To account for the burn severity of a particular fire, we use the delta *NBR* (*dNBR*) to assess how the fire changed vegetation conditions from a pre-fire baseline. *dNBR* is defined as:

(2) $$dNBR={NBR}_{pre-fire}-{NBR}_{post-fire}$$

where ${NBR}_{pre-fire}$ is the *NBR* before the fire, and ${NBR}_{post-fire}$ is the *NBR* after the fire. We use the month before fire as the unburnt control (pre-fire), i.e., June, May and the month following the fire, i.e., July, as representative of the post-fire condition. Fire occurrences are identified with NAFI to find the extent and month of occurrence. A burn with a higher severity will typically have a higher *dNBR* value, while an unburnt area will have a negative *dNBR* value or a value close to zero.

The erosion pins were placed at locations with relatively small upslope contributing areas (calculated from the 1 m spatial resolution DEM). To determine the correlation between *dNBR* and erosion/deposition, *dNBR* values representative of each pin are needed. In light of this, 60 m buffers were created around each point to account for two 30 m Landsat pixels (i.e., at least one entire Landsat pixel is captured) to capture the burnt spectral signatures. When results are discussed as spatially averaged, it is the average of all of the buffer regions surrounding an erosion measurement site.

**Figure 3:**
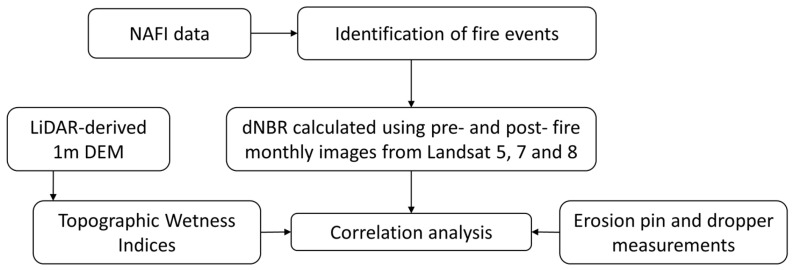
Simplified workflow chart of the study.

A simplified workflow chart is shown in Figure 3.

## 3. Results

**Figure 4:**
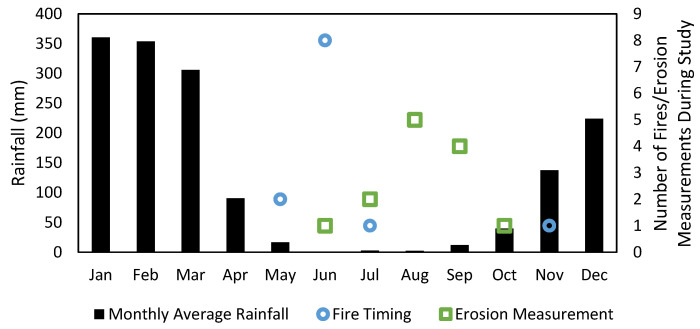
Monthly average rainfall with corresponding fire occurrences and timing of erosion measurements.

Figure 4 shows the monthly average rainfall (from 2005 to 2018) and fire timing. The monthly average rainfall shows the typical wet season occurring between November and April. All fires, with only one exception, occurred during the mid-dry season of May to July. The one exception occurred in November, which is considered as late dry season or early wet season. 

**Figure 5:**
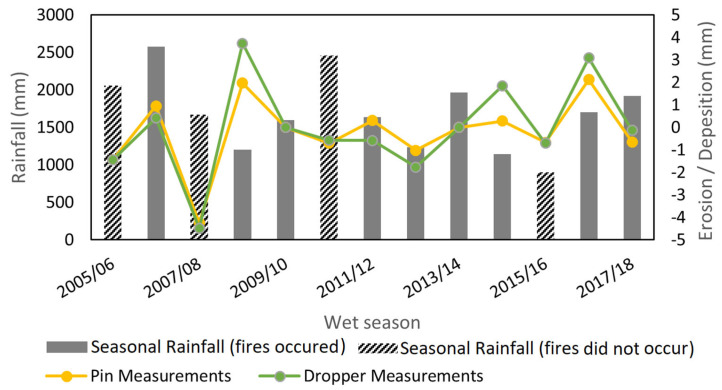
Seasonal rainfall totals and spatially averaged erosion measurements over the catchment. Columns that are completely coloured represent a season that fire occurred before the rainfall, whereas a shaded column represent a year that fire did not occur.

The catchment’s wet season rainfall for each year is plotted in Figure 5 along with catchment average erosion measurements taken from the pin and droppers. There are small differences observed in erosion measurements from the pins and droppers, although they follow the same trend. Hereafter, the average of the two measurements was used to quantify the erosion or deposition. The information detailing the occurrence of fires over the study catchment (from NAFI) was also included, which identifies when a fire occurred and its spatial extent, but not its severity. Erosion measurements were compared with the rainfall of the prior wet season (i.e., the wet season that would produce the erosion being measured) and the fire that occurred before that wet season. Figure 5 implies no pattern or relationship between the above variables. 

The average of all erosion measurements taken over the study period gave a net erosion of −0.1 mm, indicating the site is relatively stable. Erosion rates of 2–13 t ha^−1^y^−1^, (0.013–0.86 mm y^−1^) have been determined using the fallout environmental radioisotope caesium-137 (^137^Cs) in this area [52]. Surface erosion rates for the area of 0.062–0.088 mm y^−1^ have been revised using stream sediment data in the general region [53]. The erosion rates observed here are at the lower end of the reported values of Hancock et al. [52] and Wasson et al. [53]. However, the data here were determined from 21 single points across a catchment, with regular spacing that does not represent a complete hillslope. 

**Figure 6:**
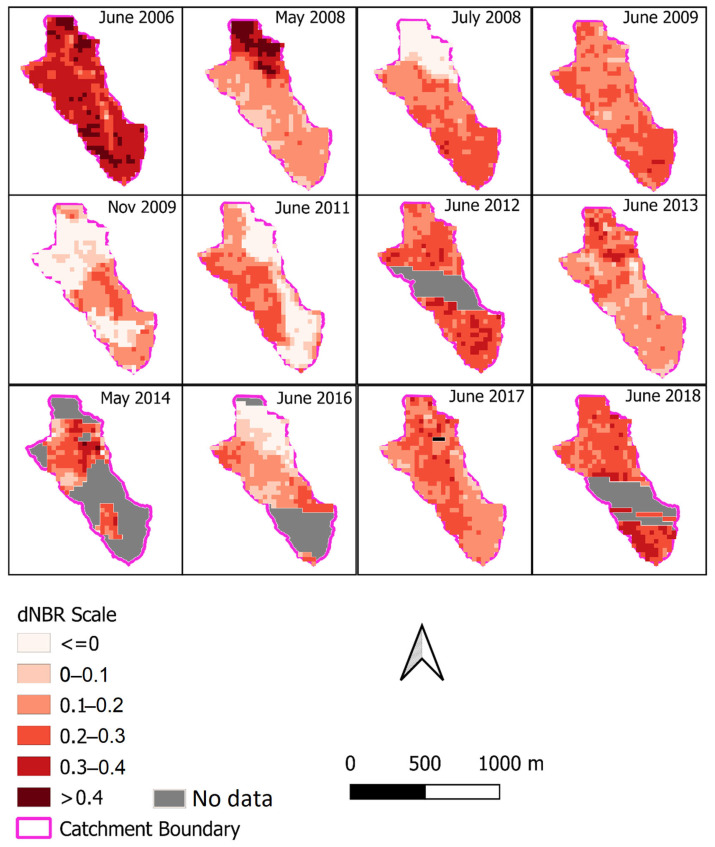
Tin Camp Creek Catchment with the Landsat derived *dNBR* (30 m resolution) for the months with fire identified by NAFI. Note during years with two fires (2008 and 2009), there was a high *dNBR* value in the first fire followed by a dramatically reduced fire severity. Blank white patches in the catchment represent areas with no data available. The locations of erosion pins are presented in Figure 1.

The *dNBR* was calculated from the months identified as pre- and post-fire. The timing of fires was identified with the NAFI data. This means that a fire in June was calculated with the *NBR* of May as pre-fire and July as post-fire. The *dNBR* for the months that NAFI identified fires is shown in Figure 6. The higher the *dNBR* value, the more severe the burn was in that location. Values of *dNBR* over +0.1 can be considered as being burnt, this value was described by Rahman et al. [54] and is consistent with values presented for interpreting *dNBR* values by UN-SPIDER/USGS (https://un-spider.org/advisory-support/recommended-practices/recommended-practice-burn-severity/in-detail/normalized-burn-ratio, accessed on 15 July 2021). There are also missing data (due to cloud cover) for certain years as the Landsat constellation did not observe that part of the catchment in a pre- and post-fire condition for the *dNBR* calculation. During years when two fires occurred over the catchment, the second fire showed a significantly reduced fire severity (see 2008 and 2009 in Figure 6 as examples). This pattern was observed on a sub-catchment spatial scale, particularly noticeable in 2008 over the northern part of the catchment. In May 2008, the north of the catchment had a severe fire; then, in July, the fire was observed as being less severe compared to the rest of the catchment. The similarities between the July 2008 and November 2009 fire *dNBR* (i.e., the second fire in a year) indicate that despite the timing in the wet/dry season cycle, the occurrence of a prior fire is the major driver of severity. There also appears to be a link between years, with regions of high-severity fires causing less severe fires the next year (see Figure 6, northern part of catchment). This is postulated to be due to fuel content not having time to replenish. Comparing this to the *dNBR* of other years (e.g., June 2013), when there was only one fire for the year, the *dNBR* showed less extremes over the catchment. 

Table 1 displays the resulting correlation when comparing the erosion measurements across the catchment and the *dNBR* of the fire event that occurred in the previous year. In these comparisons, rainfall across the catchment has impacted each point more or less the same, and therefore the influence of localised rainfall impacts is assumed to be minimised. To better understand the influence of rainfall on erosivity, indices such as EI30 can be used, but this would require recording gauges at each erosion pin. When the distance between the pins is considered, it is unlikely to have a significant difference in rainfall intensity between them. Plotting the relationships between erosion and *dNBR* suing linear regression provided similar relationships in all the years, and therefore, the plots are not presented here. Similar comparisons using catchment average erosion measurements and averages of *dNBR* values within 60 m (i.e., 2 Landsat pixels) of each erosion measurement point provided similarly poor results. In all of these comparisons, results remained the same irrespective of if burnt sites were excluded or not. 

Seasonal rainfall patterns and rainfall between years can influence erosion potential. When comparing the erosion and deposition measurements with seasonal rainfall across the catchment, there was no relationship observed. Therefore, the changes in rainfall were deemed to not significantly affect the results of this comparison. However, recording rain gauges can provide better insights if the catchment area is larger. 

Further comparison was performed by analysing changes in erosion through time at individual sites. This was compared against the *dNBR* value surrounding the same pin/dropper location. Table 2 shows a summary of all data of all the comparisons over the study catchment. There are differences in the available data due to remote sensing data gaps, no fires occurring or no erosion measurements in 2010 or 2014 (See Table 1). In almost all cases, the calculated correlation value was low, indicating no relationship. As non-burnt points were removed, the correlation changed at all the sites. However, in some of these cases, there were insufficient data to establish a meaningful relationship due to fewer fire occurrences (i.e., only two or three points—see Sites 17, 20 and 21) (Figure 1). Site 15 displays a high correlation when comparing with all sites, or just burnt sites. This appears to be an outlier in the data and goes against the trend of most sites observed across this catchment. 

Finally, (while not presented here) a comparison was made between the erosion measurement and the topographic wetness index (TWI) and SAGA Wetness Index (SWI) derived from the 1 m LiDAR DEM. This provided no significant relationship with the erosion measurements as presented in the Supplementary Materials Table S1.

## 4. Discussion

### 4.1. dNBR and Surface Erosion

The results presented in Section 3 suggest that there is little to no relationship between fire and surface erosion in this study catchment. There are some sites presented in Table 2 (e.g., Sites 15 and 19) that show a relationship with four or more points and a burnt only correlation coefficient over 0.7, although these are an exception to the trend observed across the sites. Contrary to these results, studies from other parts of Australia have shown erosion increases after fires occur. In southeast Australia, this is well documented [9,10,11,13,55,56,57]. These comparisons are from areas with different geology, climate (especially the wet dry seasonality experienced in the tropics) and vegetation communities. 

A potential influence on the lack of erosion signal observed is the presence of rocks on the surface (see Figure 2), which may act as an armour for the soil. Studies have shown the long-term impacts of fires on increasing soil stoniness in the Mediterranean [58], which, in the present study, may be causing indirect negative feedback on erosion and causing the catchment to be stable to sediment transport. Further, the rainfall patterns in southeast Australia are not as starkly different as the wet and dry seasons observed in the Northern Territory (Figure 4). After a fire occurs in southeast Australia, it is much more likely that significant rainfall will occur before vegetation has any chance of recovery. In the Northern Territory, typical fire regime there can be months after a fire before a significant precipitation event occurs leading to erosion. During this lag time, vegetation can show signs of sprouting from small amounts of moisture, leading to a reduced impact on erosion. 

There are also biogenic factors that add complexity to this environment. Hancock et al. [32] also demonstrated the introduction of surface roughness by feral pigs as a factor reducing erosion in this environment. There is also considerable on ground tree debris from a tropical cyclone in 2006 (Cyclone Monica). This tree throw event and resultant debris has been shown to influence erosion patterns and rate in this environment [59,60]. Further, Heimsath et al. [38] suggested variations in erosion rates across different landscape features, with implications for soil resource sustainability under different erosional regimes. They have also demonstrated the importance of coupling point-specific erosion rate measurements with catchment-averaged rates to gain a better understanding of landscape denudation. Specifically, a nested sampling scheme has been employed by them to quantify average erosion rates ranging from the first-order upland catchments to the main, sixth-order channel of Tin Camp Creek. They revealed that the low erosion rates (~5 m/Ma) observed in the main channel sediments reflect contributions from slowly eroding stony highlands, whereas the channels draining the study area reflected local soil production rates (~10 m/Ma off the rocky ridge; ~20 m/Ma from the soil-mantled regions). 

Most of the burns observed in this study returned relatively low *dNBR* values, indicating a low fire severity. Rahman et al. [54] found using a *dNBR* threshold of +0.1 was adequate for classifying burnt areas, which, in this study, would successfully identify burnt regions even with low fire severity (also refer to UN-SPIDER/USGS values for *dNBR* interpretation referred to early in this paper). The fires in the region surrounding the study sites have been described as being ground-only fires with biomass having low contents of combustible oils, resulting in low-severity fires [29]. The fires in the area were described by Werner and Peacock [31] as typically only burning the ground level vegetation and occasionally scorching tree canopies (also see Figure 2). Due to this description and the low burn severities observed, the majority of fires in this area may be better compared to prescribed burns in other locations (i.e., southeast Australia) rather than wildfires. It is worth noting, there are late dry season fires that can burn the canopies that are exceptions to these typical conditions described by Werner and Peacock [31]. 

Cawson et al. [12] summarised a number of studies that include both surface runoff and erosion across a range of international locations and vegetation types. While most studies have identified some change in their measured variables, some did not. The few that did not included mixed conifer forests in Idaho, USA [61], and South Carolina, USA [62,63], as well as a mixed pine-oak forest in Tennessee and Georgia, USA [64]. The reasons given for the lack of change observed in these studies, which relates to the present paper, included ‘low fire severity’, ‘low rainfall following the burn’ and ‘burn patchiness’. A similar phenomenon is seen here with low fire severity, occasional patchy burning observed while performing fieldwork and typically a long period between a fire and significant rainfall (i.e., typically after a fire in June there is little to no rainfall until November, see monthly rainfall averages in Figure 4, while ground cover may have begun to sprout changing erosion processes).

### 4.2. Environmental Implications and Fire Frequency

Shakesby et al. [13] stated that previous reports have found dry sclerophyll eucalypt forests in southeast Australia have a relatively high fire frequency that corresponds to a natural recurrence interval of as little as 13 years. The open dry sclerophyll forests in the Tin Camp Creek Catchment, as observed from the available NAFI data, burn approximately every 1–2 years. This is approximately equal to on-the-ground vegetation conditions observed during fieldwork by Hancock et al. [32] and Hancock and Lowry [33]. This is a significant increase in fire frequency compared to the typical conditions experienced in southeast Australia. However, it is worth noting that fire severity is reported to be less severe in the north of Australia compared to the southeast [65]. Additionally, there are studies that have found as invasive grass species (such as Gamba Grass) become more prevalent in northern Australia, this is likely to increase fuel loads and fire severity, which should be considered in broader landscape management [66]. There are presently no imported grass species in the study site.

Multiple publications have discussed a burning frequency of the Alligator River region surrounding the study site, including recommendations of 2–4 years or 3–5 years to promote a healthy ecosystem [29,31]. The frequency experienced in the present study catchment is much higher than this. The frequency of fires is an important aspect in allowing the building of ecosystem resilience [67]. A concerning finding is that frequent fire regimes have been shown to decrease soil carbon stores in ecosystems such as the one studied [68] and others [69]. Richards et al. [70] found an optimal fire regime of one low-severity fire every 5 years driving the biggest storage of carbon in the Northern Territory tropical savannas. The same trend of soil carbon decrease from fires occurring too frequently is found in the wet *sclerophyll* forests of southeast Queensland, Australia [71]. The currently observed fire frequency is much higher than recommended in literature for an optimal fire regime for ecosystem sustainability or soil carbon storage in the region. Hancock et al. [72] found the study site to have low soil carbon concentration. This may be hindering long-term ecosystem resilience.

### 4.3. Remote Sensing and Future Considerations

The role of remote sensing in detecting changes in ecosystems from fire is pivotal. It provides a continuous observation system that provides a spatial context to the fire’s impact and vegetation recovery time [73,74]. As new satellites are launched, or more ratios or indices are developed, there may be further improvements to the remote sensing methodologies available to natural resource managers. This will likely be in the form of spatial and temporal resolution increases. As future satellite missions become operational with improvement technology, hyperspectral remote sensing may provide solutions to assist in the management of fire [75]. 

Remote sensing has demonstrated tremendous potential for environmental monitoring. Its usefulness will continue to evolve. Satellites such as Sentinel-2 can provide datasets at a higher spatial resolution compared to Landsat for the recent years. However, there is still a need for on-ground field-based measurements. The erosion dataset at this site is one of the few long-term studies globally (i.e., data being collected for more than ten years). This type of field-based data is required to both provide insights into field scale processes as well calibrate and validate remotely-sensed data. The field-based measurements in this study provide a fundamental insight into a landscape system that is very much understudied. 

A focus of the work at this study site has been to gather information to support rehabilitation efforts of a reconstructed post-mining landform. The data from this study are from a natural environment, which has been described as being analogous to the proposed rehabilitated landforms of nearby mining sites [35]. The transferability of these results to a post-mining landform with a very different surface composition (both soil and vegetation) is open to speculation. To achieve certainty, site specific field data and validation are required. Nevertheless, while providing important data on a natural system, further research is required to investigate the findings here. 

## 5. Conclusions

The results from this study found that fire/burn severity measured from satellite observations did not correlate well with subsequent erosion severity. This finding is the opposite of the generally accepted idea that fire increases erosion. Evidence from previous literature provide reasoning for our observed results as fires in our study area were typically of low severity, had patchy burning or had a long timeframe between fires and significant rainfall. The relatively low measurements of erosion at the sites may also mean that the site was likely at an erosional equilibrium as a result of the regular removal of vegetation by fire. Year-to-year erosion and deposition can be quite high (~7 mm); however, the average erosion over the study period is quite low. These fire and rainfall characteristics are typical of the study region located in the Northern Territory, Australia. The observed fire frequency was higher than the suggested optimal recurrence interval for ecosystem health found in the literature, which may be impacting ecosystem resilience. It is worth noting that in a scenario that a late dry season fire occurs (i.e., early October), there could be significant erosion from a storm soon after. 

## Figures and Tables

**Table 1:** Pearson’s correlation coefficient (r) between each erosion/deposition measurement and corresponding *dNBR* across the study catchment throughout the years. The erosion measurements were compared to the *dNBR* of a fire in the previous year (i.e., after the seasonal rainfall has impacted the catchment). Results from relationships using all measurement locations from Figure 1 and only the point identified as burnt are provided.

Year	r	No. of Points	% of Points Burnt	Burnt Only r	Seasonal Rainfall (mm)
2006	no fire detected by NAFI				2060
2007	0.26	21	100%	0.26	2577
2008	no fire detected by NAFI				1669
2009	0.31	21	100%	0.31	1201
2010	no erosion measurements				1596
2011	no fire detected by NAFI				2457
2012	0.22	21	100%	0.22	1637
2013	0.17	17	82%	0.14	1238
2014	no erosion measurements				1963
2015	0.33	12	100%	0.33	1142
2016	no fire detected by NAFI				898
2017	0.30	14	100%	0.30	1702
2018	0.17	21	52%	0.66	1919

**Table 2:** Pearson’s correlation coefficient (r) calculated when comparing erosion/deposition values and the *dNBR* values of the individual sites through time. Presented is a comparison of all available data and then a subset of only the years when fire was identified by NAFI.

Site ID	r	No. of Years Available	Burnt Only r	No. of Years Site Was Burnt
1	0.20	6	0.17	5
2	0.39	6	0.60	5
3	0.10	6	0.22	5
4	0.10	7	0.17	6
5	0.20	7	0.00	6
6	0.17	7	0.41	5
7	0.33	7	0.20	5
8	0.20	7	0.00	5
9	0.77	6	0.57	4
10	0.62	6	0.47	4
11	0.46	6	0.62	3
12	0.49	6	0.51	3
13	0.70	7	0.57	4
14	0.20	7	0.17	4
15	0.78	6	0.94	5
16	0.10	7	0.32	3
17	0.40	6	0.92	3
18	0.14	7	0.33	3
19	0.51	7	0.88	4
20	0.59	5	1	2
21	0.30	5	1	2

## Data Availability

Remote sensing data are available for loading and analysis from Digital Earth Australia. Erosion measurement data can be requested from the corresponding author.

## Supplementary Materials

The following supporting information can be downloaded at: https://www.mdpi.com/article/10.3390/s24072282/s1, Digital Earth Australia—Data Loading Query; Table S1: R^2^ calculated between the SWI and TWI against the erosion/deposition from every year.

## References

[1] Earl N., Simmonds I. (2018). Spatial and Temporal Variability and Trends in 2001–2016 Global Fire Activity. J. Geophys. Res. Atmos..

[2] Hill R.S., Jordan G.J. (2016). Deep history of wildfire in Australia. Aust. J. Bot..

[3] Peters M.P., Iverson L.R., Matthews S.N., Prasad A.M. (2013). Wildfire hazard mapping: Exploring site conditions in eastern US wildland–urban interfaces. Int. J. Wildland Fire.

[4] Fernandes P.M., Botelho H.S. (2003). A review of prescribed burning effectiveness in fire hazard reduction. Int. J. Wildland Fire.

[5] Peters M.P., Iverson L.R. (2017). Incorporating fine-scale drought information into an eastern US wildfire hazard model. Int. J. Wildland Fire.

[6] Shrestha A., Grala R.K., Grado S.C., Roberts S.D., Gordon J.S. (2021). Landowner Concern about Wildfires and Implementation of Fuel Reduction Treatments. J. For..

[7] van der Werf G.R., Randerson J.T., Giglio L., van Leeuwen T.T., Chen Y., Rogers B.M., Mu M., van Marle M.J.E., Morton D.C., Collatz G.J. (2017). Global fire emissions estimates during 1997–2016. Earth Syst. Sci. Data.

[8] Keeley J.E. (2009). Fire intensity, fire severity and burn severity: A brief review and suggested usage. Int. J. Wildland Fire.

[9] Atkinson G. (2012). Soil Erosion Following Wildfire in Royal National Park, NSW. Proc. Linn. Soc. N. S. Wales.

[10] Yang X., Zhu Q., Tulau M., McInnes-Clarke S., Sun L., Zhang X. (2018). Near real-time monitoring of post-fire erosion after storm events: A case study in Warrumbungle National Park, Australia. Int. J. Wildland Fire.

[11] Smith H.G., Dragovich D. (2008). Post-fire hillslope erosion response in a sub-alpine environment, south-eastern Australia. Catena.

[12] Cawson J.G., Sheridan G.J., Smith H.G., Lane P.N.J. (2012). Surface runoff and erosion after prescribed burning and the effect of different fire regimes in forests and shrublands: A review. Int. J. Wildland Fire.

[13] Shakesby R.A., Wallbrink P.J., Doerr S.H., English P.M., Chafer C.J., Humphreys G.S., Blake W.H., Tomkins K.M. (2007). Distinctiveness of wildfire effects on soil erosion in south-east Australian eucalypt forests assessed in a global context. For. Ecol. Manag..

[14] Parsons A., Robichaud P.R., Lewis S.A., Napper C., Clark J.T. (2010). Field Guide for Mapping Post-Fire Soil Burn Severity.

[15] Chiang S.-H., Ulloa N.I. (2019). Mapping and tracking forest burnt areas in the indio maiz biological reserve using sentinel-3 SLSTR and VIIRS-DNB imagery. Sensors.

[16] García M.J.L., Caselles V. (1991). Mapping burns and natural reforestation using thematic mapper data. Geocarto Int..

[17] Christopoulou A., Mallinis G., Vassilakis E., Farangitakis G.P., Fyllas N.M., Kokkoris G.D., Arianoutsou M. (2019). Assessing the impact of different landscape features on post-fire forest recovery with multitemporal remote sensing data: The case of Mount Taygetos (southern Greece). Int. J. Wildland Fire.

[18] Li L., Xin X., Zhao J., Yang A., Wu S., Zhang H., Yu S. (2023). Remote Sensing Monitoring and Assessment of Global Vegetation Status and Changes during 2016–2020. Sensors.

[19] Andela N., Morton D.C., Giglio L., Paugam R., Chen Y., Hantson S., van der Werf G.R., Randerson J.T. (2019). The Global Fire Atlas of individual fire size, duration, speed and direction. Earth Syst. Sci. Data.

[20] Sánchez Sánchez Y., Martínez Graña A., Santos-Francés F. (2021). Remote Sensing Calculation of the Influence of Wildfire on Erosion in High Mountain Areas. Agronomy.

[21] Efthimiou N., Psomiadis E., Panagos P. (2020). Fire severity and soil erosion susceptibility mapping using multi-temporal Earth Observation data: The case of Mati fatal wildfire in Eastern Attica, Greece. Catena.

[22] Argentiero I., Ricci G.F., Elia M., D’Este M., Giannico V., Ronco F.V., Gentile F., Sanesi G. (2021). Combining methods to estimate post-fire soil erosion using remote sensing data. Forests.

[23] Mallinis G., Maris F., Kalinderis I., Koutsias N. (2009). Assessment of post-fire soil erosion risk in fire-affected watersheds using remote sensing and GIS. GIScience Remote Sens..

[24] Pérez-Cabello F., Montorio R., Alves D.B. (2021). Remote sensing techniques to assess post-fire vegetation recovery. Curr. Opin. Environ. Sci. Health.

[25] Baghbani A., Choudhury T., Costa S., Reiner J. (2022). Application of artificial intelligence in geotechnical engineering: A state-of-the-art review. Earth-Sci. Rev..

[26] Howe A.A., Parks S.A., Harvey B.J., Saberi S.J., Lutz J.A., Yocom L.L. (2022). Comparing Sentinel-2 and Landsat 8 for burn severity mapping in Western North America. Remote Sens..

[27] Kurbanov E., Vorobev O., Lezhnin S., Sha J., Wang J., Li X., Cole J., Dergunov D., Wang Y. (2022). Remote sensing of forest burnt area, burn severity, and post-fire recovery: A review. Remote Sens..

[28] Russell-Smith J., Edwards A.C., Sangha K.K., Yates C.P., Gardener M.R. (2020). Challenges for prescribed fire management in Australia’s fire-prone rangelands–the example of the Northern Territory. Int. J. Wildland Fire.

[29] Hernandez-Santin L., Erskine P.D., Bartolo R.E. (2020). A review of revegetation at mine sites in the Alligator Rivers Region, Northern Territory, and the development of a state and transition model for ecological restoration at Ranger uranium mine. J. Clean. Prod..

[30] Andersen A.N., Braithwaite R.W., Cook G.D., Corbett L.K., Williams R.J., Douglas M.M., Gill A.M., Setterfield S.A., Muller W.J. (1998). Fire research for conservation management in tropical savannas: Introducing the Kapalga fire experiment. Aust. J. Ecol..

[31] Werner P.A., Peacock S.J. (2019). Savanna canopy trees under fire: Long-term persistence and transient dynamics from a stage-based matrix population model. Ecosphere.

[32] Hancock G., Lowry J., Dever C., Braggins M. (2015). Does introduced fauna influence soil erosion? A field and modelling assessment. Sci. Total Environ..

[33] Hancock G.R., Lowry J.B.C. (2015). Hillslope erosion measurement-a simple approach to a complex process. Hydrol. Process..

[34] Hancock G.R., Lowry J.B.C., Dever C. (2016). Surface Disturbance and Erosion by Pigs: A Medium Term Assessment for the Monsoonal Tropics. Land Degrad. Dev..

[35] Uren C. (1992). An Investigation of Surface Geology in the Alligator Rivers Region for Possible Analogues of Uranium Mine Rehabilitation Structures.

[36] Story R., Galloway R.W., McAlpine J.R., Aldrick J.M., Williams M.A.J. (1976). Lands of the Alligator Rivers Area, Northern Territory.

[37] Saynor M.J., Erskine W.D., Evans K.G., Eliot I. (2004). Gully initiation and implications for management of scour holes in the vicinity of the Jabiluka Mine, Northern Territory, Australia. Geogr. Ann..

[38] Heimsath A.M., Fink D., Hancock G.R. (2009). The ‘humped’soil production function: Eroding Arnhem Land, Australia. Earth Surf. Process. Landf..

[39] Russell-Smith J., Ryan P.G., Durieu R. (1997). A LANDSAT MSS-Derived Fire History of Kakadu National Park, Monsoonal Northern Australia, 1980-94: Seasonal Extent, Frequency and Patchiness. J. Appl. Ecol..

[40] Bonta M., Gosford R., Eussen D., Gerguson N., Loveless E., Witwer M. (2017). Intentional Fire-Spreading by “Firehawk” Raptors in Northern Australia. J. Ethnobiol..

[41] Harris S., Tapper N., Packham D., Orlove B., Nicholls N. (2008). The relationship between the monsoonal summer rain and dry-season fire activity of northern Australia. Int. J. Wildland Fire.

[42] Setterfield S.A., Rossiter-Rachor N.A., Douglas M.M., McMaster D., Adams V., Ferdinands K. The impacts of Andropogon gayanus (gamba grass) invasion on the fire danger index and fire management at a landscape scale. Proceedings of the Nineteenth Australasian Weeds Conference.

[43] Setterfield S.A., Rossiter-Rachor N.A., Douglas M.M., Wainger L., Petty A.M., Barrow P., Shepherd I.J., Ferdinands K.B. (2013). Adding fuel to the fire: The impacts of non-native grass invasion on fire management at a regional scale. PLoS ONE.

[44] Setterfield S.A., Rossiter-Rachor N.A., Hutley L.B., Douglas M.M., Williams R.J. (2010). Biodiversity Research: Turning up the heat: The impacts of Andropogon gayanus (gamba grass) invasion on fire behaviour in northern Australian savannas. Divers. Distrib..

[45] Dhu T., Dunn B., Lewis B., Lymburner L., Mueller N., Telfer E., Lewis A., McIntyre A., Minchin S., Phillips C. (2017). Digital earth Australia–unlocking new value from earth observation data. Big Earth Data.

[46] Tarboton D.G. (1997). A new method for the determination of flow directions and upslope areas in grid digital elevation models. Water Resour. Res..

[47] Conrad O., Bechtel B., Bock M., Dietrich H., Fischer E., Gerlitz L., Wehberg J., Wichmann V., Böhner J. (2015). System for Automated Geoscientific Analyses (SAGA) v. 2.1.4. Geosci. Model Dev..

[48] Bretreger D., Yeo I.Y., Melchers R. (2021). Terrain wetness indices derived from LiDAR to inform soil moisture and corrosion potential for underground infrastructure. Sci Total Environ..

[49] Loughran R.J. (1989). The measurement of soil erosion. Prog. Phys. Geogr..

[50] Flood N. (2014). Continuity of Reflectance Data between Landsat-7 ETM+ and Landsat-8 OLI, for Both Top-of-Atmosphere and Surface Reflectance: A Study in the Australian Landscape. Remote Sens..

[51] Key C.H., Benson N. (2006). Landscape Assessment (LA) Sampling and Analysis Methods.

[52] Hancock G., Loughran R., Evans K., Balog R. (2008). Estimation of soil erosion using field and modelling approaches in an undisturbed Arnhem Land catchment, Northern Territory, Australia. Geogr. Res..

[53] Wasson R., Saynor M., Lowry J. (2021). The natural denudation rate of the lowlands near the Ranger mine, Australia: A target for mine site rehabilitation. Geomorphology.

[54] Rahman S., Chang H.-C., Hehir W., Magil C., Tomkins K. Inter-Comparison of Fire Severity Indices from Moderate (Modis) and Moderate-To-High Spatial Resolution (Landsat 8 & Sentinel-2A) Satellite Sensors. Proceedings of the IGARSS 2018—2018 IEEE International Geoscience and Remote Sensing Symposium.

[55] Atkinson G. (1984). Erosion Damage Following Bushfires. J. Soil Conserv..

[56] White I., Wade A., Worthy M., Mueller N., Daniell T., Wasson R. (2006). The vulnerability of water supply catchments to bushfires: Impacts of the January 2003 wildfires on the Australian Capital Territory. Australas. J. Water Resour..

[57] Tomkins K.M., Humphreys G.S., Wilkinson M.T., Fink D., Hesse P.P., Doerr S.H., Shakesby R.A., Wallbrink P.J., Blake W.H. (2007). Contemporary versus long-term denudation along a passive plate margin: The role of extreme events. Earth Surf. Process. Landf..

[58] Shtober-Zisu N., Wittenberg L. (2021). Long-term effects of wildfire on rock weathering and soil stoniness in the Mediterranean landscapes. Sci. Total Environ..

[59] Hancock G., Evans K.G., McDonnell J., Hopp L. (2012). Ecohydrological controls on soil erosion and landscape evolution. Ecohydrology.

[60] Hancock G., Lowry J. (2021). Quantifying the influence of rainfall, vegetation and animals on soil erosion and hillslope connectivity in the monsoonal tropics of northern Australia. Earth Surf. Process. Landf..

[61] Arkle R.S., Pilliod D.S. (2010). Prescribed fires as ecological surrogates for wildfires: A stream and riparian perspective. For. Ecol. Manag..

[62] Galang M.A., Morris L.A., Markewitz D., Jackson C.R., Carter E.A. (2010). Prescribed burning effects on the hydrologic behavior of gullies in the South Carolina Piedmont. For. Ecol. Manag..

[63] Richter D., Ralston C., Harms W. (1982). Prescribed fire: Effects on water quality and forest nutrient cycling. Science.

[64] Elliot K.J., Vose J.M. (2005). Initial effects of prescribed fire on quality of soil solution and stream water in the southern Appalachian Mountains. South. J. Appl. For..

[65] Gill A.M., Moore P.H.R., Williams R.J. (1996). Fire weather in the wet-dry tropics of the World Heritage Kakadu National Park, Australia. Aust. J. Ecol..

[66] Rossiter N.A., Setterfield S.A., Douglas M.M., Hutley L.B. (2003). Testing the grass-fire cycle: Alien grass invasion in the tropical savannas of northern Australia. Biodivers. Res..

[67] Gillson L., Whitlock C., Humphrey G. (2019). Resilience and fire management in the Anthropocene. Ecol. Soc..

[68] Pellegrini A.F.A., Ahlstrom A., Hobbie S.E., Reich P.B., Nieradzik L.P., Staver A.C., Scharenbroch B.C., Jumpponen A., Anderegg W.R.L., Randerson J.T. (2018). Fire frequency drives decadal changes in soil carbon and nitrogen and ecosystem productivity. Nature.

[69] Wright J., DeLaMater D., Simha A., Ury E., Ficken C. (2020). Changes in Prescribed Fire Frequency Alter Ecosystem Carbon Dynamics. Ecosystems.

[70] Richards A.E., Cook G.D., Lynch B.T. (2011). Optimal Fire Regimes for Soil Carbon Storage in Tropical Savannas of Northern Australia. Ecosystems.

[71] Muqaddas B., Zhou X., Lewis T., Wild C., Chen C. (2015). Long-term frequent prescribed fire decreases surface soil carbon and nitrogen pools in a wet sclerophyll forest of Southeast Queensland, Australia. Sci. Total Environ..

[72] Hancock G.R., Murphy D., Evans K.G. (2010). Hillslope and catchment scale soil organic carbon concentration: An assessment of the role of geomorphology and soil erosion in an undisturbed environment. Geoderma.

[73] White J.D., Ryan K.C., Key C.C., Running S.W. (1996). Remote Sensing of Forest Fire Severity and Vegetation Recovery. Int. J. Wildland Fire.

[74] Hammill K.A., Bradstock R.A. (2006). Remote sensing of fire severity in the Blue Mountains: Influence of vegetation type and inferring fire intensity. Int. J. Wildland Fire.

[75] Veraverbeke S., Dennison P., Gitas I., Hulley G., Kalashnikova O., Katagis T., Kuai L., Meng R., Roberts D., Stavros N. (2018). Hyperspectral remote sensing of fire: State-of-the-art and future perspectives. Remote Sens. Environ..

