# Supplementary material for: The Impacts of Burn Severity and Frequency on Erosion in Western Arnhem Land, Australia
Source: Sensors (Basel). 2024 Apr 3;24(7):2282. doi: 10.3390/s24072282 (PMC11014083; doi:10.3390/s24072282)
Supplement: Supplementary file 1 [file sensors-24-02282-s001.zip › sensors-2921201-supplementary.pdf]

Supplementary Information

Digital Earth Australia – Data Loading Query

The query used to load Landsat data from Digital Earth Australia is presented below to allow better reproducibility of the results in this article:

```
query = {
    'geopolygon': geom,
    'time': ('2005-01-01', '2018-21-31'),
    'measurements': ['nbart_nir', 'nbart_swir_2'],
    'output_crs': 'EPSG:3577',
    'resolution': (30, 30),
    'group_by': 'solar_day'
```

NOTE: the variable 'geom' is the catchment shapefile projected in EPSG:3577 which is GDA94 Australian Albers.

For the present study, this query was used to load analysis ready data products:

- ga_ls5t_ard_3
- ga_ls7e_ard_3
- ga_ls8c_ard_3

With corrections applied to the Landsat 8 product as per the text in Section 2.4 in the main body of the article.

Supplementary Terrain Analysis Results

Table S1 shows the coefficient of determination (R^2) of the relationship between SWI and TWI against the erosion measurements for each year.

Table S1. R^2 calculated between the SWI and TWI against the erosion/deposition from every year.

Year	SWI	TWI
**2006**	0.0008	0.0243
**2007**	0.0001	0.0067
**2008**	0.0458	0.0128
**2009**	0.0063	0.0403
**2010**	n/a	n/a
**2011**	0.0028	0.0001
**2012**	0.0074	0.0008
**2013**	0.0124	0.0113
**2014**	n/a	n/a
**2015**	0.0008	0.0008
**2016**	0.0595	0.0004
**2017**	0.0385	0.0205
**2018**	0.0179	0.0015
